# Body Composition and a School Day Hydration State among Polish Children—A Cross-Sectional Study

**DOI:** 10.3390/ijerph17197181

**Published:** 2020-09-30

**Authors:** Agnieszka Kozioł-Kozakowska, Beata Piórecka, Agnieszka Suder, Paweł Jagielski

**Affiliations:** 1 Department of Pediatrics, Gastroenterology and Nutrition, Institute of Pediatrics, Faculty of Medicine, Jagiellonian University Medical College, Wielicka Str. 265, 30-663 Kraków, Poland; 2 Department of Nutrition and Drug Research, Institute of Public Health, Faculty of Health Science, Jagiellonian University Medical College, Grzegórzecka Str. 20, 31-531 Kraków, Poland; beata.piorecka@uj.edu.pl (B.P.); paweljan.jagielski@uj.edu.pl (P.J.); 3 Department of Anatomy, Institute of Basic Sciences, Faculty of Motor Rehabilitation, University of Physical Education in Cracow, Jana Pawła II Av. 78, 31-571 Kraków, Poland; agnieszka.suder@poczta.fm

**Keywords:** hydration status, body composition, fat tissue, children

## Abstract

Background: Little is known on the relationship between obesity and hydration level in children. To explore the possible association between children’s hydration status and body composition, we conducted this cross-sectional study. Methods: The survey was carried out in 2018 in Preliminary and High Schools from the Malopolska Province, Poland. The study group consisted of 264 children aged 7–15 years. The level of hydration was assessed based on urine osmolality during a school day. The examined had anthropometric tests and body composition assessment (FM, BF%, FFM, TBW, TBW%). Odds ratio (OR) and 95% confidence interval (CI) were calculated using a logistic regression analysis. Results: In the study group, 9.5% of the examined were overweight, 7.2% obese, and it referred more to the country than towns (*p* < 0.05). Improper hydration was found in 53% of children, and 16.3% of them were severely dehydrated during a school day (urine osmolality > 1000 mOsm/kgH_2_O). The level of dehydration was higher in children with excessive body fat (BF%) than in children with normal BF% [903.00 vs. 775 mOsm/kgH_2_O]. Older age (>10 y) showed inverse association with dehydration [OR 0.52 (95% CI; 0.28–0.99)] and excessed BF% showed 2.3-fold increase in odds of dehydration during a school day [OR 2.39 (95% CI; 1.15–4.94)]. Improper hydration was a risk factor of difficulties with concentration declared by students during a school day OR 2.85 (95% CI; 1.16–6.99). Conclusions: Attention should be paid to appropriate hydration especially in children with excessive body fat content who feature a higher risk of dehydration and fluid demand.

## 1. Introduction

The condition of young people’s health in Poland is subject to constant and systematic changes. New health problems arise due to alterations in their way of life as well as social and environmental changes. These problems include overweight and obesity [1]. In the Polish population 1/3 of eight-year-olds feature excessive weight (COSI study), and among adolescents (11–15 years), according to the HBSC (Health Behaviour School-Aged Children) study, 16.6% were overweight and 4.7% obese [2,3]. Recent studies point to another important aspect of children’s health connected with obesity issue, which is insufficient hydration. Children are at greater risk of dehydration, since their regulatory mechanisms are still insufficient compared to adults; voiding volume and frequency reach their full maturity by adolescence [4]. New study results revealed that today total body water (TBW) in children is lower than in the past because of higher level of fatness. Therefore, obese children are more likely to become dehydrated [5].

The insufficient hydration is associated with kidney and metabolic diseases in adulthood as well as affect cognitive performance in children [6,7,8,9]. Unfortunately, available data show that children do not meet EFSA (European Food Standards Agency) recommendations for total fluid intake, which is for girls: 1.9 L/day and 2.12 L/day for boys [10]. Therefore, they are at risk of dehydration. Results of last cross-sectional surveys from 13 countries revealed that 61% of children and 75% of adolescents did not consume enough water from fluids than recommended by the EFSA guidelines [11]. Another review showed that among the 19 countries that reported comparison of water/fluid intake with guidelines, 60 ± 24% of children failed to meet them [12].

To assess hydration status urinary biomarkers such as urine osmolality, urine specific gravity, and urine color can be used. Specifically, osmolality is helpful in determining changes in cell hydration and renal response to fluid intake [13]. The available literature suggests that many children have highly concentrated urine, indicating insufficient fluid intake [14,15,16,17,18]. Only a few studies so far examined association between hydration status and obesity in children. [15,19]. To the best of our knowledge, there are no studies that assessed the level of hydration in Polish children depending on body composition. Therefore, the aim of this study was to assess the prevalence of dehydration and its association with body composition in a sample of Polish children, as well as risk factors linked with this condition. Based on the literature review, we hypothesize that children with excess body fat have higher urinary osmolality and higher risk of dehydration.

## 2. Material and Methods

The study was designed as a cross-sectional survey, comprising 264 children aged 7–15, carried out in 2018 in selected preliminary and high schools from the city of Krakow and neighborhood, 46.2% urban dwellers and 53.8% countryside ones. The questionnaire was used to assess families’ socio-economic status (place of residence, parents’ educational level) and the declared health problems, which could be associated with dehydration such as: difficulties with concentration, headaches, frequency of urination at school, feeling thirsty, somnolence, and difficulty in remembering. We also asked about physical activity level, and depending on the answers, the group was divided into 2 subgroups: children with ordinary physical activity (attending PE classes and 0–2 h a week of additional physical activities) and extraordinary physical activity (attending PE classes and more than 3 h a week of additional physical activities). The questionnaires were filled in by children and their parents. Participation in the study was voluntary, the informed consent was signed by the children’s legal guardians. The following inclusion criteria were established: healthy children who do not take oral medications on a regular basis and do not follow a special diet, informed consent of parents and the child to participate in research. The following exclusion criteria were established: chronic kidney disease, heart disease, diabetes treated with insulin, lack of consent. The protocol was conducted according to the ethical principles stated in the Helsinki Declaration (1964). All procedures involving research study participants were approved by the Bioethical Committee of the Jagiellonian University. Written informed consent was obtained from all subjects no. 122.6120.320.2016.

### 2.1. Assessment of Nutritional Status

The examined children had anthropometric tests, i.e., body mass was measured with medical weight (standardized to 0.1 kg) without shoes and in light clothes, and body height was measured in Frankfurt Plane position with a height meter within an accuracy of 0.1 cm. Waist circumference was measured with an accuracy of 0.5 cm standing up, after gentle exhalation, as the minimum circumference measurable on the horizontal plane between the lowest part of the chest and iliac crest. The WHtR (waist-to-height ratio) indicator was also used with the value of 0.5 demonstrating prevalence of abdominal obesity [20]. WHtR was calculated according to the formula: waist circumference-waist (cm)/height (cm). The same researcher measured height, weight, and waist circumference. Body mass index (BMI) was computed according to the following formula: BMI = weight (kg)/height^2^ (m^2^). BMI-Z score (BMI-z) was calculated and interpreted in relation to national percentile charts [21]. The body composition was measured by bioimpedance (BIA) using Tanita analyzer (Tanita BC-418 Tanita Corporation, Tokyo, Japan). The measurements were performed according to the manufacturer’s guidelines at least 2 h after the ingestion of a light breakfast and urination. The following data were collected: fat mass (FM) (kg), body fat percentage (BF%), fat-free mass (FFM) (kg), total body water (TBW) (kg), and total body water percentage of weight (TBW%). An individual was classified by BF% as “normal” or “excessive” according to international guidelines for children [22].

### 2.2. Hydration Status

In the assessment of hydration, a urine sample given during school stay (after breakfast) were collected. Based on urine osmolality (Uosm) the study group was divided into three groups such as: 300–800 mOsm/kgH_2_O—proper hydration, 800–1000 mOsm/kgH_2_O—mild dehydration, >1000 mOsm/kgH_2_O—severe dehydration [23].

### 2.3. Statistical Analysis

All the collected data were analyzed statistically with the use of the PS IMAGO PRO 6 (IBM SPSS Statistics 26) software. Mann–Whitney U-test or Kruskal–Wallis test were used to compare variables not normally distributed between groups. If the Kruskal–Wallis test was significant, a post-hoc analysis was performed to determine which groups differ from each other group. Chi-square test was used to check association between categorical variables, *p*-value less than 0.05 was considered an indication of a statistically significant result. The risk factors of dehydration depending on anthropometric parameters and body composition were assessed in logistic regression model (OR) with 95% confidence interval (CI). Odds ratios were adjusted for possible contributions from other variables in the model. Association between the level of hydration and declared health problems also was checked in a logistic regression model.

## 3. Results

There were 264 students participating in the survey—143 (54.2%) boys and 121 (45.8%) girls. The median of age was 10.08 (7.03–14.03) years. There were no differences in the age and gender of respondents depending on place of residence. Fathers of children living in the city had higher education level *p* < 0.01 (Table 1).

In the study group, 76% of the children had normal BMI, 9.5% were overweight, 7.2% were obese, and the same percentage of children had BMI below norm. Anthropometric measurements including selected parameters of BIA are shown in Table 2. There were statistically significant differences in the anthropometric and bioimpedance parameters between groups of boys and girls due to WHtR (*p* < 0.05), FM (*p* < 0.05), and BF% (*p* < 0.01), which were higher in the group of girls than boys. Boys had significantly higher percentage of TBW than girls (66.57% vs. 60.82%) considering body weight, but there were not any differences in total body water (TBW) in kilograms. Children from the countryside featured significantly higher anthropometric parameters (weight, waist circumference, BMI, BMI-z score) as well as body composition (FM, BF%, TBW%).

The median value of urine osmolality of the whole study group was 825 (82–1404) mOsmol/kg of water. In the study group, 47% of children were properly hydrated, 36.7% slightly dehydrated, and 16.3% of children were severely dehydrated during a school day. No gender differences were observed. The medians of urine osmolality were shown in Table 3. In reference to anthropometric parameters and body composition depending on hydration level, significant differences were observed. BMI z-score, FM, BF% were significantly higher in children with a higher level of dehydration. Inverse dependence was observed for the TBW% that decreased with increasing dehydration (Table 3).

**Figure 1:**
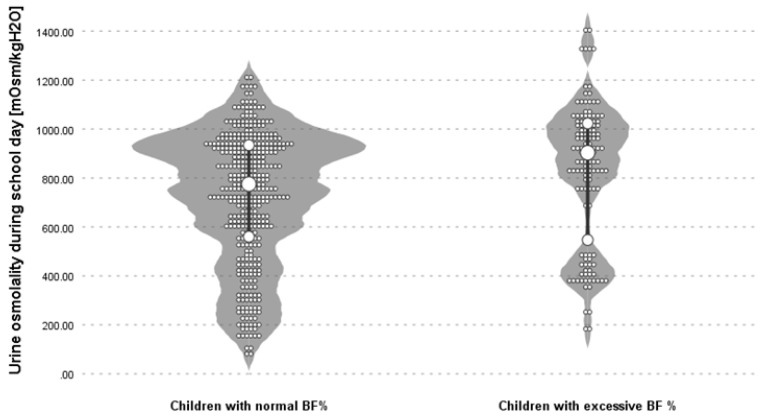
Distribution of results urine osmolality depending on the interpretation of percentage of body fat (BF%) in the study group.

The value of urine osmolality was higher in children with excessive BF% [903.00 mOsm/kgH_2_O (176–1404.00)] than in children with normal BF% [775 mOsm/kgH_2_O (82.00–1211.00)], *p* < 0.01 (Figure 1). Children with excess BF% were statistically significantly dehydrated during a school day (*p* < 0.01), 28.8% of children with excessive fat content vs. 12% with normal fat content had urine osmolality over 1000 mOsm/kgH_2_O, which means they were severely dehydrated during their stay at school.

The potential predictors of children’s hydration were checked (Table 4). Due to the use of logistic regression analysis, the study group was divided into two: properly hydrated and dehydrated children. Only two predictors were statistically significant (age and BF%). More younger children (60.7% vs. 46.90%) and overfat children were dehydrated (66.7% vs. 46.6%). No differences between groups depending on the level of parents’ education, place of residence, the level of physical activities, BMI, BMI-z score, or WHtR were observed. The combined effect of two significant variables (age and BF%), which were related with the level of hydration was investigated using logistic regression (Table 5). The analysis confirmed that BF% and age are significant risk factors of dehydration. Older age (>10 y) showed inverse association with dehydration [OR 0.52 (95% CI;0,28–0.99)], and excess BF% showed 2.39-fold increase in odds of dehydration during a school day [OR 2.39 (95% CI; 1.15–4.94)].

Taking into consideration declared health problems such as: difficulties in concentration, headaches, frequency of urination at school, feeling thirsty, somnolence, difficulty in remembering connected with dehydration, the logistic regression model was calculated. We observed that inadequate hydration was a risk factor of difficulties with concentration during school OR 2.85 (95% CI; 1.16–6.99).

## 4. Discussion

Healthy eating is one of the key actions for obesity prevention, and healthy fluid consumption is a part of a balanced diet. Recently, a study has shown that the promotion of healthy hydration in elementary schools, by increasing water accessibility through water fountains and providing lessons to promote water consumption, was an efficient strategy to lower the risk of being overweight by 31% in the interventional group [24]. In addition, this is so important when we look at data showing that incidence of children obesity has increased two–threefold in the last two decades [25]. In the OLAF study, representative for the Polish population, 18.6% of boys and 14.5% of girls within the age of 7–18 years were either overweight or obese. In Poland, this problem is most common among children living in cities with over 500,000 inhabitants (especially when both parents work) and in the country (agricultural regions) [21]. The prevalence of overweightness and obesity in this study is consistent with the results from the general population. Differences between boys and girls reflects more the natural difference of body composition between genders than differences of obesity occurrences because no significant differences were observed due to the BMI and BMI-z score between sex groups. Improving water intake is increasingly considered as a priority action for healthier lifestyle in children. The WHO Regional Office for Europe has prepared the key components of effective policies and provides advice on a complex action plan that could be implemented in Poland. One of the action areas comprises a suggestion to restrict the availability of sugar-sweetened beverages (SSBs) on the premises of educational establishments [26]. Although research does not confirm that increased water consumption reduces body weight, it is clearly evident that replacing water with SSBs show inverse association with weight loss [27].

The results of clinical studies confirmed that many children have highly concentrated urine before or during school, which can suggest that a large proportion of children arrive at school already slightly dehydrated and deepen this state by not drinking adequately during a school day [16,17,19,27,28]. Based on 24 urine osmolality in the NHANES (National Health and Nutrition Examination Survey) study (children aged 6–19), 54.5% of children were inadequately hydrated [28]. In a study of 529 French children aged 9–11 years, the hydration status in the morning was assessed using the urine osmolality collected after breakfast, and almost two thirds of the examined showed a hydration deficit in the morning, despite having breakfast [16]. In addition, in Greek children aged 9–13 years, 44% of boys and 23% of girls were dehydrated at schools [28]. An even higher proportion of dehydration was observed in Belgium and Egypt where, respectively, 76% and 80% of children were inappropriately hydrated during a school day [15,17]. Results from the Liq.In surveys demonstrate that many schools simply do not provide access to water for children, which has consequences in drinking behaviors [11]. In Poland in 2014, about 98% of the population had access to water from municipal water supplies with a quality in accordance with the requirements specified in the decision of the Minister of Health on potable water quality. Unfortunately, over 60% of Polish people do not trust the water supply and are afraid to consume it without boiling. Furthermore, it is not without significance the fact that water must also be acceptable from an organoleptic standpoint. Polish children can buy bottle water in school shops or in vending machines where unfortunately the water competes with other sweetened drinks [29].

Among boys and younger children higher urine density was more frequently observed [16,17,19,27,28]. It might be due to changes in proportion of lean and fat tissue connected with puberty and a decrease in water % in lean tissue with a different change rate in boys and girls [4]. In the literature, there is also a suggestion that the lower urine osmolality in girls might be caused by a higher water density of the ingested food (ml/ kcal) and a lower insensible water loss (ml/kcal) in girls than boys. However, higher osmolality in boys could not be explained by a lower fluid intake in boys, since we have not found any sex differences in fluid intake in the literature [30,31]. In our study younger children were also more dehydrated, but statistically insignificant differences were observed only between sexes. Sex also was not a risk factor of dehydration in logistic regression analysis, which is not compatible with data from literature but can result from a smaller study group in comparison to the discussed studies. EFSA guidelines for daily water intake per kg body weight are as follows: 56 mL/kg/day, 44 mL/kg per day, and 39 mL/kg per day in boys and girls 4 to 8 years old, in boys 9 to 13 years old, and in girls 9 to 13 years old, respectively [11]. That is because human water needs depend on body surface area and a higher body surface to body mass ratio, namely higher insensible water loss through the skin may add to dehydration condition in obese children. Additionally, BMI is based on higher energy requirements, greater food consumption, and higher metabolic production, therefore water turnover rates increase with BMI [32]. Studies confirm that obese children were less hydrated than normal weight children. In Maffeis et al., hydration status was not satisfactory in 34% of obese and 20% of normal-weight children [19]. Association between BMI and hydration in children (osmolality) is observed, but not between weight and height separately [17,19,33]. In our study children with higher BMI-z score had higher urine osmolality, but no differences depending on weight or height were observed. BMI is not the best indicator for assessing childhood obesity; it does not distinguish between lean and fat mass and cannot completely identify children who may suffer from health issues related to excess adipose tissue. Moreover, the BF% estimated by BIA had almost perfect reproducibility, making it an applicable research tool in studies that investigate body composition changes at different times [34]. In Belgian children with higher BF%, lower hydration (over-day osmolality) was found [15]. According to the results of the own study, severe dehydration was more common in overfat children, and the urine osmolality was significantly higher in children with excessive BF% (*p* < 0.05). A higher percentage of obese children in the countryside may also be the reason for the differences in hydration levels depending on the place of residence in the study group.

Insufficient hydration can affect not only physical health (renal dysfunction, constipation) but also mental health. Even slight dehydration negatively affects the mental condition. It leads to an increased feeling of fatigue and reduces mental efficiency by decreasing memory, attention, and response time. It reduces visual and motor coordination being especially important during the learning process [7,9,35,36]. Fadda et al. examined in the RTC study the effect of drinking water on cognitive performance, fatigue, and vigor in school children. Based on urine osmolality measurement, 84% of children were dehydrated (morning Uosm >800 mOsm/kgH_2_O) at the start of a school day. Drinking water had beneficial effects on memory, verbal reasoning, and vigor [37].The results of new study of Drozdowska et al. suggest that a water friendly environment supports school-aged children in adequate water intake resulting in better cognitive performance, especially short-term memory [6]. In our study, 53% of all children featured an inadequate level of hydration during their stay at school, which turned out to be a risk factor of difficulties with concentration declared by students and could affect both current and long term learning outcomes.

In conclusion, the results of this study showed that more than a half of the examined children were not sufficiently hydrated during a school day. The analysis confirmed that BF% and younger age are significant risk factors of dehydration. It is important to constantly emphasize the role of proper hydration for children, especially children with excess body fat.

Potential limitations of this study are as follows: the cross-sectional design that does not allow for exploring the cause–effect relationship between variables but just their degree of association; narrow recruitment area restricted to southern Poland that does not allow extending the results to all populations; no fluid intake assessment; using one study sample for assessing hydration status. It would be better to collect more than one sample during a school day, but in practice this is hard to perform. Collecting even one sample was problematic, the children were ashamed to donate a sample, they had difficulties with urinating into the container (especially younger children), they forgot to urinate. Some of the children did not use the toilet at all during their school stay, which is consistent with the observations from other studies, which highlighted that up to one out of four children report not using the toilets at school to urinate [38,39].

## Figures and Tables

**Table 1:** Socioeconomic characteristic of the study group.

Parameter	Total (%) N = 264	Village (%) *n* = 142	Town (%) *n* = 122	*p*-Value
Gender				
Boys	54.2	53.9	53.7	NS ^a^
Girls	45.8	46.1	46.3	
Age				
7–9	44.3	44.0	45.5	NS ^a^
10–14	55.7	56.0	54.5	
Mother’s Education				
Primary	0.4	0.7	-	
Vocational	10.0	10.1	9.3	
Secondary	30.5	30.9	29.7	NS ^b^
Higher	59.1	58.3	61.0	
Father’s Education				
Primary	0.4	0.7	-	
Vocational	22.4	29.7	13.9	
Secondary	31.9	31.9	32.2	<0.01 ^b^
Higher	45.3	37.7	53.9	

^a^ = result chi-square test, ^b^ = result Mann–Whitney U-test, NS = not statistically significant.

**Table 2:** The body composition and anthropometric parameters in the study group.

Parameters	Total	Boys	Girls	*p* Value	Town	Village	*p* Value
Age (years)	10.10 (7.03–14.03)	10.10 (7.03–13.77)	10.15 (7.83–14.03)	NS	10.09 (7.03–13.98)	10.11 (7.48–14.03)	NS
Weight [kg]	35.00 (20.30–70.80)	34.50 (21.89–70.80)	36.00 (20.30–59.00)	NS	33.40 (20.30–64.30)	37.00 (21.80–70.80)	<0.05
Height [cm]	1.42 (1.20–1.75)	1.41 (1.20–1.73)	1.44 (1.21–1.75)	NS	1.42 (1.20–1.72)	1.44 (1.20–1.75)	NS
BMI [kg/m^2^]	17.20 (12.57–30.54)	17.26 (12.20–28)	17.04 (12.57–30.54)	NS	16.65 (13.20–26.09)	17.97 (12.57–30.54)	<0.05
BMI-z score	−0.03 (−1.60–4.59)	−0.12 (−1.60–3.15)	0.02 (−1.52–4.59)	NS	−0.25 (−1.60–2.82)	0.11 (−1.52–4.59)	<0.05
Waist (cm)	61.00 (48.00–92.00)	61.00 (48.00–92.00)	60.00 (48.00–86.00)	NS	60.00 (50.00–83.00)	63.00 (48.00–92.00)	<0.01
WHtR	0.43 (0.35–0.62)	0.43 (0.37–0.62)	0.42 (0.35–0.60)	<0.05	0.42 (0.36–0.59)	0.43 (0.35–0.62)	<0.05
Fat mass [kg]	7.35 (3.40–27.50)	6.60 (3.40–27.50)	8.30 (3.40–25.30)	<0.05	6.40 (3.40–19.80)	8.40 (3.60–27.50)	<0.05
BF%	21.47 (11.60–42.88)	19.23 (11.60–42.77)	22.93 (14.73–42.88)	<0.01	19.44 (11.60–37.50)	22.93 (13.51–42.88)	<0.01
Fat-Free Mass [kg]	27.60 (6.00–50.60)	27.50 (6.00–50.60)	27.90 (16.50–44.90)	<0.01	27.75 (6.00–46.50)	27.20 (11.20–50.30)	NS
Total Body Water [kg]	23.50 (12.70–39.00)	23.60 (13.10–39.00)	23.20 (12.70–33.00)	NS	23.45 (12.70–36.70)	23.30 (12.70–38.80)	NS
Total Body Water [% of body weight]	63.33 (40.90–83.62)	66.57 (42.12–83.62)	60.82 (40.90–78.64)	<0.01	65.69 (46.5–83.62)	59.62 (40.90–83.28)	<0.01

BMI = body mass index, BF% = percentage of body fat, data are shown as median (min–max), *p* = Mann–Whitney U-test, NS = not statistically significant.

**Table 3:** The body composition and anthropometric parameters in the study depending on level of hydration.

Parameters	Total	Proper hydration (A)	Mild Dehydration (B)	Severe Dehydration (C)	*p* Value
Age (years)	10.10 (7.03–14.03)	10.00 (7.00–14.00)	9.00 (7.00−14.00)	10.00 (7.00–13.00)	NS
Weight [kg]	35.00 (20.30–70.80)	34.65 (20.60–67.00)	34.90 (20.30–68.70)	38.40 (21.80–70.80)	NS
Height [cm]	1.42 (1.20–1.75)	1.43 (1.20–1.75)	1.43 (1.20–1.73)	1.44 (1.23–1.68)	NS
BMI [kg/m^2^]	17.20 (12.57–30.54)	17.03 (12.57–30.54)	16.99 (13.14–28.96)	18.72 (12.81–25.43)	NS
BMI-z score	−0.03 (−1.60–4.59)	−0.18 (−1.52–4.59) ^C^	0.01 (−1.60–3.15)	0.28 (−1.43–2.82) ^A^	<0.05
Waist (cm)	61.00 (48.00–92.00)	60.00 (48.00–85.00)	60.00 (48.00–92.00)	62.00 (48.00–88.00)	NS
WHtR	0.43 (0.35–0.62)	0.43 (0.35–0.59)	0.43 (0.37–0.62)	0.44 (0.37–0.60)	NS
Fat mass [kg]	7.35 (3.40–27.50)	7.25 (0.80–24.50) ^C^	7.45 (1.10–23.40)	9.20 (3.40–24.50) ^A^	<0.05
BF %	21.47 (11.60–42.88)	20.80 (13.74–42.88)	21.96 (11.60–42.77)	22.77 (13.36–42.88)	<0.05
Fat-Free Mass [kg]	27.60 (6.00–50.60)	28.40 (6.00–45.10)	27.20 (11.20–50.60)	28.30 (18.10–50.30)	NS
Total Body Water [kg]	23.50 (12.70–39.00)	23.80 (12.70–35.90)	22.35 (13.10–39.00)	23.60 (13.18–33.80)	NS
Total Body Water [% of body weight]	63.33 (40.90–83.62)	64.45 (46.27–82.62) ^C^	63.07 (42.12–83.62)	59.09 (40.90–75.00) ^A^	<0.05
Level of urine osmolality [mOsm/kgH_2_O]	825.00 (82.00–1404.00)	551.5 (82.00–800.00) ^B,C^	914.00 (804.00–997.00) ^A,C^	1062.00 (1007.00–1404.00) ^A,B^	<0.01

BMI = body mass index, BF% = percentage of body fat, levels of dehydration: 300–800 mOsm/kgH_2_O—proper hydration, 800–1000 mOsm/kgH_2_O—mild dehydration, >1000 mOsm/kgH_2_O—severe dehydration; data are shown as median (min–max), *p* = Kruskal-Wallis test, ^A,B,C^ = differences between groups, NS = not statistically significant.

**Table 4:** Potential predictors of dehydration in studied group.

Predictors	% of Adequately Hydrated Children	% of Inadequately Hydrated Children	*p*-Value
Gender			
Girls	50.4	49.2	NS
Boys	44.1	55.9	
Age			
7–9	39.3	60.7	<0.05
10–14	62.9	49.3	
Place of residence			
Town	52.1	47.9	NS
Village	42.6	57.4	
Mother’s Education			
Vocational	53.8	46.2	NS
Secondary	43.0	57.0	
Higher	47.1	52.9	
Father’s Education			
Vocational	50.9	49.1	NS
secondary	48.1	51.9	
Higher	43.5	56.5	
Level of Physical Activities			
Ordinary	45.4	54.6	NS
Extraordinary	48.9	51.1	
BMI [kg/m^2^]			
Underweight	52.6	47.4	NS
Normal	48.3	51.7	
Overweight	44.0	56.0	
Obesity	31.6	68.4	
BMI-z score			
Underweight	33.3	66.7	NS
Normal	49.0	51.0	
Overweight	39.5	60.5	
Obesity	37.5	62.5	
WHtR			
Proper	47,8	52.2	NS
Visceral obesity	40.6	59.4	
BF%			
Normal	53.4	46.6	<0.05
Overfat	33.3	66.7	

NS = not statistically significant.

**Table 5:** Risk factors of dehydration during school day in studied group.

Outcome	Predictor Variables	Unadjusted OR	Adjusted OR	(95% CI)
Dehydration	Age (10–14)	0.57	0.52	0.28–0.99
	BF% (Overfat)	2.29	2.39	1.15–4.94
